# The effects of curcumin supplementation on high-sensitivity C-reactive protein, serum adiponectin, and lipid profile in patients with type 2 diabetes: A protocol for systematic review and meta-analysis

**DOI:** 10.1097/MD.0000000000031942

**Published:** 2022-12-09

**Authors:** Jie Li, Lifang Sun

**Affiliations:** a Department of Clinical Laboratory, First People’s Hospital of Tianshui, Gansu, China; b Department of Blood Transfusion, First People’s Hospital of Tianshui, Gansu, China.

**Keywords:** blood glucose, curcumin, meta-analysis, type 2 diabetes mellitus

## Abstract

Type 2 diabetes mellitus (T2DM) is an ensemble of metabolic diseases that has reached pandemic dimensions all over the world. There is a lack of evidence on the contribution of curcumin in the treatment of T2DM. We conducted a protocol for systematic review and meta-analysis to evaluate whether curcumin supplementation is effective and safe in T2DM patients.

**Methods:**

The systematic review will follow the guidelines of Preferred Reporting Items for Systematic Reviews and Meta-analyses Protocols (PRISMA-P). We will obtain studies through PubMed, Cochrane Library, Embase, Web of Science, and Medline databases. In addition, we will also collect 4 databases of China: China National Knowledge Infrastructure, China Biomedical Literature Database, China Science Journal Database, and Wan-fang Database. Eligible study conference abstracts and reference lists of manuscripts will be searched. The data collection and analysis will be conducted independently by 2 reviewers. Meta-analysis will be performed using Review Manager software, version 5.3 (Update Software Ltd, Oxford, Oxon, UK).

**Results:**

The results of this systematic review and meta-analysis will be published in a peer-reviewed journal.

**Conclusion:**

The findings of this systematic review may encourage supplementation of curcumin and its preparation specifically in T2DM patients. Nevertheless, the application of curcumin supplementation in clinical practice should be taken with individual’s contributing factors.

## 1. Introduction

Type 2 diabetes mellitus (T2DM) is an ensemble of metabolic diseases that has reached pandemic dimensions all over the world.^[1–3]^ Diabetes has reached pandemic dimensions, affecting over 400 million people worldwide, and it is also becoming relevant in developing countries.^[4,5]^ Among the huge and heterogeneous number of patients with diabetes, T2DM represents the most prevalent form. From the Global Burden of Diseases study (2016), it emerged that T2DM and its complications were responsible for the 22% increase in disability in the last 10 years, impacting significantly on public health.^[6]^ The multifactorial nature of the pathology makes patient management, which includes lifelong drug therapy and lifestyle modification, extremely challenging.^[7,8]^ It is well-known that T2DM is a preventable disease, therefore lowering the incidence of new T2DM cases could be a key strategy to reduce the global impact of diabetes.

Currently, there is growing evidence on the efficacy of the use of medicinal plants supplements for T2DM prevention and management. Among these medicinal plants, curcumin is gaining a growing interest in the scientific community.^[9,10]^ Curcumin is a bioactive molecule present in the rhizome of the Curcuma longa plant, also known as turmeric.^[11]^ Curcumin has different pharmacological and biological effects that have been described by both in vitro and in vivo studies, and include antioxidant, cardioprotective, anti-inflammatory, antimicrobial, nephroprotective, antineoplastic, hepatoprotective, immunomodulatory, hypoglycemic and antirheumatic effects. In animal models, curcumin extract delays diabetes development, improves β-cell functions, prevents β-cell death, and decreases insulin resistance.^[12,13]^

Regarding diabetes, an animal study highlighted that curcumin and its analogues have a mechanism of action similar to that of thiazolidinedione, an antidiabetic drug, through peroxisome proliferator-activated receptor-γ activation. Thus, curcumin may be effective in the regulation of glycemia and lipidaemia.^[14]^

However, there is a lack of evidence on the contribution of curcumin in the treatment of T2DM. In this study, we conducted a protocol for systematic review and meta-analysis to evaluate whether curcumin supplementation is effective and safe in T2DM patients.

## 2. Methods

### 2.1. Study registration

This protocol has been registered on international prospective register of systematic review (PROSPERO) as CRD42022368820. It will be conducted following the guideline of Preferred Reporting Items for Systematic Reviews and Meta-analyses Protocols (PRISMA-P).^[15]^ No ethical approval is needed for this type of systematic review as the data is reviewed retrospectively.

### 2.2. Criteria for including studies in this review

Studies were considered eligible if they met the following criteria: patients aged 40–70 years old, diagnosed with T2DM (based on criteria of the American Diabetes Association and WHO); intervention group received curcumin supplementation; control group received placebo; outcome measures included at least one of the following: high-sensitivity C-reactive protein level, serum adiponectin level, and lipid profile; randomized controlled trials.

The exclusion criteria for this study were as follows: duplicate studies, experience summaries, case reports, reviews; studies with incomplete information or animal experiments; studies with fewer than 15 cases; the loss rate of the subjects was > 20%.

### 2.3. Search strategy

**Figure 1.**
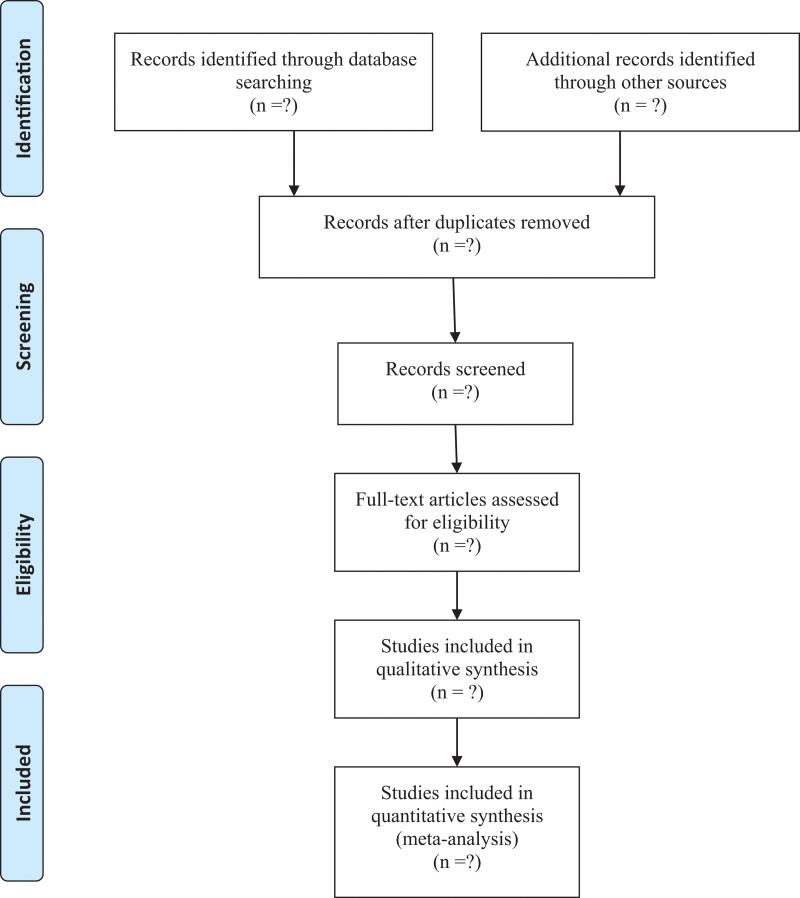
Flow diagram of study selection.

This study will use the PubMed, Cochrane Library, Embase, Web of Science, and Medline databases. In addition, we will also collect 4 databases of China: China National Knowledge Infrastructure, China Biomedical Literature Database, China Science Journal Database, and Wan-fang Database. We will consider articles published between database initiation and October 2022. Two authors will independently draft and carry out the search strategy. In addition, we manually retrieve other resources, including the reference lists of identified publications, conference articles, and gray literature. The key terms used for the search are “Type 2 diabetes,” “curcumin” and “randomized.” Two authors independently review all titles and abstracts of studies identified by the above searches. Full texts of any potentially useful studies are reviewed, and disagreements are resolved by discussion. Table 1 provides the search strategies in the PubMed database, and other databases will use these strategies similarly. Study selection will be performed in accordance with the Preferred Reporting Items for Systematic Reviews and Meta-Analyses flowchart (Fig. 1).

**Table 1:** Search strategy in PubMed.

Number Search terms
#1 curcum[Ti/Ab]
#2 curcumin extract[Ti/Ab]
#3 curcuma extract[Ti/Ab]
#4 turmeric[Ti/Ab]
#5 turmeric extract[Ti/Ab]
#6 curcuminoid[Ti/Ab]
#7 turmeric yellow[Ti/Ab]
#8 diferuloylmethane[Ti/Ab]
#9 #1 OR #2 OR #3 OR #4 OR #5 OR #6 OR #7 OR #8
#10 type 2 diabetes mellitus[Ti/Ab]
#11 pathoglycemia[Ti/Ab]
#12 hyperglycemia[Ti/Ab]
#13 insulin resistance[Ti/Ab]
#14 glycuresis[Ti/Ab]
#15 #10 OR #11 OR #12 OR #13 OR #14
#16 #9 AND #15

### 2.4. Data extraction

The following data are extracted for each article: bibliographical data, including authors and year of publication; clinical trial features such as sample size, study flow, recruitment method, criteria for inclusion and exclusion, primary measures, time and point of assessments, and duration of the intervention; participant characteristics such as age, sex, and so on; patient background, including country and race; and study drop-out rate and handling of missing data.

### 2.5. Risk of bias assessment

Two investigators will separately assess the risk of bias of the included studies using the Cochrane risk of bias assessment tool.^[16]^ The evaluation of each study mainly included the following 7 aspects: random sequence generation, allocation hiding, blinding of participants and personnel, blinding of outcome assessment, incomplete outcome data, incomplete outcome data, selective outcome reporting, and other biases. Finally, the bias of the study will be rated on 3 levels: “low,” “high,” and “ambiguous.” Discrepancies will be addressed by consulting a third reviewer.

### 2.6. Statistical analysis

Statistical heterogeneity is explored using a chi-square test and expressed as an I^2^ index (a significance level of *P* < .10). *I*^2^ values over 50% represented substantial heterogeneity. If there is no heterogeneity, a fixed-effects model is used for meta-analysis; otherwise, a random-effects model is used. Continuous variables are expressed as the weighted mean difference and 95% confidence interval. Potential publication bias is tested with a funnel plot. To evaluate publication bias, we will construct a funnel plot if the number of included studies is sufficient.^[17]^ All analyses are performed with Review Manager software, version 5.3 (Update Software Ltd, Oxford, Oxon, UK).

### 2.7. Summary of evidence

The assessment of evidence for all outcomes will be summarized using the Grading of Recommendations Assessment, Development and Evaluation (GRADE) approach.^[18]^ The quality of evidence will be rated as high, moderate, low, and very low quality.

### 2.8. Dealing with missing data

We will try to contact the corresponding author of any study with missing data to obtain the missing information. If the corresponding author cannot be reached, we will use the available data for synthesis, and the potential impact of the missing information will be reviewed.

## 3. Discussion

Curcumin is an active component of turmeric and has a polyphenolic structure. Due to its anti-inflammatory and antioxidant properties, it has attracted increasing attention for the prevention and treatment of diabetes mellitus.^[19]^ The findings of this systematic review may encourage supplementation of curcumin and its preparation specifically in T2DM patients. Nevertheless, the application of curcumin supplementation in clinical practice should be taken with individual’s contributing factors. In spite of the biological and therapeutic effects of curcumin, the plasma and tissue content of this compound is very low due to its low biological stability, rapid metabolism, and systemic elimination from the body. Recently, a kind of curcumin with nanorange formulation was reported and significantly improved the therapeutic effects of curcumin. This compound was known as the “nanocurcumin” and suggested using from this compound in future studies.

## Author contributions

Write manuscript and data analysis: Jie Li.

Review and edit: Lifang Sun.
